# Authenticated communication from quantum readout of PUFs

**DOI:** 10.1007/s11128-017-1649-0

**Published:** 2017-07-05

**Authors:** Boris Škorić, Pepijn W. H. Pinkse, Allard P. Mosk

**Affiliations:** 1 Department of Mathematics and Computer Science, Eindhoven University of Technology, P.O. Box 513, 5600 MB Eindhoven, The Netherlands; 2 Complex Photonic Systems (COPS), MESA+ Institute for Nanotechnology, University of Twente, P.O. Box 217, 7500 AE Enschede, The Netherlands; 3 Debye Institute for Nanomaterials Science, Utrecht University, P.O. Box 80.000, 3508 TA Utrecht, The Netherlands

**Keywords:** Physical unclonable function, PUF, Authentication, Quantum readout

## Abstract

Quantum readout of physical unclonable functions (PUFs) is a recently introduced method for remote authentication of objects. We present an extension of the protocol to enable the authentication of *data*: A verifier can check if received classical data were sent by the PUF holder. We call this modification QR-d or, in the case of the optical-PUF implementation, QSA-d. We discuss how QSA-d can be operated in a parallel way. We also present a protocol for authenticating quantum states.

## Introduction

### Authentication

Authentication is an essential part of human interaction and automated transactions. Before one engages in any transaction with an unknown entity, it is prudent to authenticate the other party. We distinguish between *authentication of objects* and *authentication of data*. The former is very old. The aim is to verify that an object under scrutiny is exactly the same object that has been seen before, or that it belongs to a class of approved objects. Examples are coins, security holograms, paintings and biometrics. The cat-and-mouse game of counterfeiting versus anti-counterfeiting has been going on for millennia.

In *data authentication* the aim is twofold: to make sure that the stated origin of data is correct, and to verify that the data was not manipulated after its creation. In many situations the authenticity of data is far more important than confidentiality (secrecy), e.g., monetary transactions, contracts, news, credentials, telemetry, public voting, websites. There are two main approaches. (i) Inscribe the data into an object that is hard to counterfeit and manipulate. Examples are paper money and ID cards containing high-tech authenticity marks. (ii) Use cryptography. In the case of symmetric crypto, Message Authentication Codes; in the case of asymmetric cryptography, digital signatures.

It is interesting to note that the advent of quantum computers will break [24] all currently deployed asymmetric crypto: RSA, Diffie-Hellman and Elliptic Curves [18]. When this ‘Cryptocalypse’ happens, the only leftover asymmetric crypto for authentication will be *post-quantum* schemes based on, e.g., hashes [6], lattices or error-correcting codes.^1^

Note also that quantum key distribution^2^ (QKD) [3, 4, 12, 17, 19, 23] does not solve the problem of authentication in the post-quantum world, since QKD itself requires authenticated classical communication as a starting point.

### Quantum readout of physical unclonable functions

The best known *classical* anti-counterfeiting objects (i.e., without quantum degrees of freedom) are called physical unclonable functions (PUFs) [22]. PUFs are, on technological grounds,^3^ hard to clone, and they have a highly unique challenge–response behavior when physically probed. In this paper we will not consider the use of PUFs as Physically Obfuscated Keys for secret key storage [13], but only their physical, non-cryptographic anti-counterfeiting use. The best currently known PUF technology of this type is optical PUFs. Optical PUFs are light-diffusing disordered objects that contain many non-absorbing randomly shaped scatterers in random locations. When laser light impinges on them, three-dimensional coherent multiple scattering occurs, resulting in exiting light (transmitted or reflected) that forms a highly complex speckle pattern which depends strongly on the scatterer positions as well as the characteristics of the incoming light such as angle, focus, etc. [15].

From a scientific point of view, there are very appealing aspects to PUF-based authentication. The manufacture and verification of PUFs does not depend on any trade secret, nor does any secret data have to be stored. All stored data are public and have to be protected only against manipulation. Such a ‘fighting with open visors’ approach allows one to organize competitions like those known in cryptography for standardizing ciphers. A possible drawback is the cumbersome enrollment procedure: Challenge–response pairs (CRPs) have to be measured and stored for every PUF individually.

When the verifier has full physical control over the to-be-verified object, a scenario that we refer to as *hands on*, PUF authentication is ‘perfect’ in the sense that no attack exists other than physical cloning of the PUF. This attack contradicts the security assumption. The situation is more complicated in *hands-off* scenarios, where the object is far away, or the PUF holder does not want to relinquish control over his PUF. Here typically the verifier has to rely on far-away trusted measurement devices. In the hands-off case, attacks exist on the *protocol* level, i.e., attacks on the communication or the exchanged light. The attacker either hacks or tricks the remote device: He receives PUF challenges and then feeds into the device the corresponding responses from the publicly known CRP tables. In this way he successfully authenticates without having access to the actual PUF.

A conceptual breakthrough was achieved by the introduction of *quantum readout* of PUFs [25, 26]. The security is based on the unclonability of unknown quantum states [8, 10, 11, 28]. Quantum readout needs a two-way quantum channel. The challenge is a quantum state that contains more information than what can be extracted from it by measurement. The attacker cannot accurately determine the challenge, and hence the CRP table is of no use. The verifier, on the other hand, knows precisely which response state should be generated by the PUF and can efficiently verify if the returned state matches this. Quantum readout entirely eliminates attacks at the protocol level. Quantum readout has been experimentally demonstrated using optical PUFs [16, 27]. The technique was dubbed quantum secure authentication (QSA).

The focus of QR and QSA in [16, 25] was object authentication. The possibility of data authentication was mentioned but not further explored.

### Contributions and outline

In Sect. 2 we introduce the notation and terminology used throughout the paper and briefly review quantum readout of PUFs. The contributions are as follows.

- We introduce an extension of the QR protocol which authenticates classical data sent by the PUF holder. We call it ‘QR-d.’ QR-d allows the recipient of the classical data to verify that the data were sent by the PUF holder. (Sects. 3.1, 3.2.)
- For the QSA implementation of QR, we show how a server can be set up which authenticates many messages to many verifiers in parallel. (Sect. 3.3). This setup could be orders of magnitude faster than a server that provides cryptographic signatures.
- In Sect. 3.4 we show how QSA-d can also be applied to quantum information. Bob sends a quantum state (known to himself) such that Alice is able to verify that it originated from Bob. Alice does not learn the quantum state.

## Preliminaries

### Notation and terminology

We use the standard bra-ket notation for quantum states. We distinguish between the classical description of a state and the physical state itself. The quantum state is written as $| \psi \rangle$, and its classical description is $\psi$ (without the brackets). Similarly, we distinguish between the matrix *R*, which consists of classical data, and the operator $\hat{R}$, which acts on quantum states. This is important, e.g., when we deal with a PUF whose action on quantum states is $\hat{R}$: The data *R* are publicly known and can be used for classical computations, but there exists only one object that is able to perform the operation $\hat{R}$ *losslessly* on quantum states.

### The quantum readout protocol

Quantum readout of PUFs, in its simplest form, works as follows. We consider a challenge space ${\mathcal H}$ which is a *d*-dimensional Hilbert space. A PUF is a classical object that can map a challenge $| \psi \rangle \in {\mathcal H}$ to a response $\hat{R}| \psi \rangle \in {\mathcal H}$, where $\hat{R}$ is unique for each PUF. The operator $\hat{R}$ is not necessarily unitary, as it could describe for instance only the reflection or transmission part of scattered light.

#### Attacker model

We refer to a PUF identity by its transfer matrix *R*. The main security assumption is: It is infeasible for an attacker who does not have access to a PUF *R* to perform the action $\hat{R}$ losslessly on a few-photon challenge.

#### Enrollment phase

A PUF is manufactured. Its properties are measured and then stored in a way that prevents malicious modifications. In the case of optical PUFs, the stored enrollment data could be the transfer matrix *R* describing the input-output behavior of the PUF. Alternatively, the same information can be stored in the form of a list of CRPs. The PUF is given to the prover, Bob.

#### Authentication phase

At some later time, a verifier Alice wants to check if Bob still possesses the PUF. Alice fetches the enrollment data for Bob’s PUF. She picks a random $\psi$, prepares a single particle in the state $| \psi \rangle \in {\mathcal H}$ and sends it to Bob. Bob lets the particle interact with his PUF, resulting in the response state $| \omega \rangle =\hat{R}| \psi \rangle$. He sends the response state back to Alice. Alice, knowing $\psi$ and *R*, computes $\omega =R\psi$ and performs a measurement of the projection operator $| \omega \rangle \langle \omega |$ on the returned state. She repeats the challenge–response procedure multiple times, each time with a freshly random $\psi$. If enough rounds produced a ‘1’ measurement outcome, Alice is convinced that the particles are being returned by someone who has access to Bob’s PUF.

#### Security against challenge-estimation attacks

In each round of the above protocol, challenge-estimation attacks have a probability of at most $2/(1+d)$ to cause a ‘1’. The overall probability of a false accept is exponentially small in the number of rounds. The protocol can be generalized to *n*-particle states $| \psi \rangle ^{\otimes n}$, in which case the attacker’s per-particle success probability is upper bounded by $\frac{n+1}{n+d}$ [26]. The security is based on the unclonability of unknown quantum states [8, 10, 11, 28].

## Data authentication from quantum readout

### The simplest construction

We propose an extension of QR that achieves authentication of classical data. In the enrollment phase, Bob receives *q* different PUFs labeled $0,\ldots ,q-1$. Bob wants to send an authenticated message $x=(x_j)_{j=1}^N$, $x_j\in \{0,\ldots ,q-1\}$ to Alice. They perform the following steps.

1. Bob sends *x* to Alice over a public, un-authenticated classical channel.
2. For $j\in \{1,\ldots ,N\}$ Alice and Bob run the QR protocol, using the PUF labeled $x_j$.

In each run $j\in \{1,\ldots ,N\}$, Alice is convinced that someone with access to Bob’s PUF $x_j$ is returning her challenge states. By implication, the holder of Bob’s PUFs agrees with the message *x* which Alice received over the un-authenticated channel.

The security of this protocol trivially reduces to the security of the original QR protocol.

### An alternative construction

The scheme in Sect. 3.1 requires Bob to essentially send the message *x* twice. This can be avoided, but at some cost. Consider the above protocol, but without step 1. Alice now does not know beforehand which PUF will be activated by Bob. Alice has to modify her verification equipment such that it can distinguish between *q* different authentic response states.

**Fig. 1:**
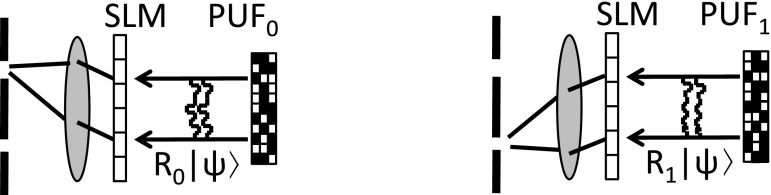
A single SLM configuration can verify *q* distinct responses. Example for $q=2$

In the optical-PUF implementation, QSA [16], there is an elegant way to achieve this functionality (see Fig. 1). The configuration of the spatial light modulator (SLM) is modified so that each of the *q* correct wavefronts is transformed into its own single mode, which is then directed through a pinhole into a detector. (While incorrect response states are transformed to random speckle which typically does not lead to a detection event.) The price to pay is that the efficiency of the SLM transform is reduced by a factor *q*, i.e., photons are lost.

One may be tempted to think that our exclusion of step 1 now enables Bob to send *secret* information *x* to Alice. However, bear in mind that Bob is presenting his choice of PUF to the whole outside world; if an attacker briefly hijacks Alice’s optical path to Bob, the attacker too can have access to Bob’s PUF and determine $x_j$.

### Massively parallel QSA-d

We observe that there are two vastly different time scales in QSA. On the one hand, the preparation of an SLM and the photodetection takes a verifier at least 20 microseconds.^4^ On the other hand, the interaction between a challenge and the prover’s PUF lasts not much longer than the duration of the challenge pulse, some 10 picoseconds.

**Fig. 2:**
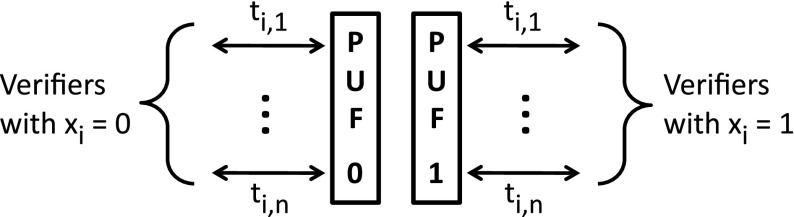
Parallel QSA-d

While one verifier is preparing his equipment for the next round, the prover has plenty of time to let other verifiers interact with his PUFs. This allows for a parallel implementation of QSA-d as shown in Fig. 2. The prover is a server that has to authenticate different messages $x^{(1)},x^{(2)},\ldots$ to many different verifiers $V_1, V_2,\ldots$. For simplicity we consider $q=2$. Extension to $q\ge 3$ is straightforward. In time slot *i* the server presents PUF$_0$ to *n* different verifiers *v* which have symbol $x^{(v)}_i=0$ and PUF$_1$ to *n* verifiers *v* which have $x^{(v)}_i=1$. Each time slot is subdivided into smaller time intervals $t_{i,1},\ldots ,t_{i,n}$. Each verifier gets his own interval.

The parallelism can be increased further if different wavelengths are included; challenges at different wavelengths can pass through a PUF simultaneously and can be routed independently.

In theory, the above-mentioned timescales would allow for an amount of parallelism $n = 20\,\upmu \mathrm{s}/10\,\mathrm{ps}=2\cdot 10^6$. In practice the bottleneck is the switching speed that can be realized using, e.g., electro-optical modulation. Currently the switching time is of the order of 1 ns. This yields $n=20\,\upmu \mathrm{s}/1\,\mathrm{ns}=2\cdot 10^4$.

### Authenticating quantum information

Consider the construction in Sect. 3.2 for $q=2$. Alice sends a random challenge state $| \psi \rangle$. Bob routs the challenge into PUF$_0$ with probability amplitude $\alpha$ and into PUF$_1$ with amplitude $\beta$. ($|\alpha |^2+|\beta |^2=1$.) The response state is

1 $$\begin{aligned} \alpha R_0| \psi \rangle +\beta R_1| \psi \rangle , \end{aligned}$$

and this is sent to Alice. Alice’s verification equipment (Fig. 1) focuses the response through the ‘0’ pinhole with amplitude $\alpha$ and through the ‘1’ pinhole with amplitude $\beta$.

The upshot is (i) that Alice has received a qubit state with parameters $\alpha ,\beta$ unknown to her (but known to Bob), and (ii) that Alice knows that this qubit state can be sent only by the holder of Bob’s PUFs. Thus, we have achieved PUF-based authentication of quantum states, with the restriction that the sender must know the quantum state.

As in Sect. 3.2, the data cannot be considered secret. An attacker could challenge Bob’s setup using a macroscopic amount of light, and the response would reveal $\alpha$ and $\beta$ with high accuracy. Alice too could use a macroscopic amount of light to learn $\alpha ,\beta$, but then she cannot trust that the response is coming from Bob.

Note that the SLM-based construction has photon losses. For $q=2$ the loss is of order 50%. There are two ways to compensate for the losses. (a) Alice’s challenges are single-photon states. The process is repeated (with fresh random $\psi$) until a response makes it through the pinholes.

(b) Alice’s challenge consists of more than one photon, e.g., *n* photons in state $| \psi \rangle ^{\otimes n}$ or a weak laser pulse as in [16]. The number of photons is scaled such that with high probability at least one photon makes it through the pinholes. The security of the authentication is guaranteed as long as *n* (or the expected *n*) is small enough [26] compared to the number of modes.

## Summary and discussion

QR as presented in [16, 25] remotely verifies the authenticity of a PUF, but does not authenticate messages. In this paper we have shown two ways to modify QR so that it can authenticate messages. Bob’s choice of PUF corresponds to a message symbol $0,1,\ldots ,q-1$ in a *q*-ary alphabet. In the first and simplest protocol, Bob first announces the message *x* so that Alice knows which PUF responses to expect. In the second protocol, Bob makes no announcement; Alice’s verification equipment needs to be able to handle more than one correct response. In the QSA implementation of the second protocol, the efficiency (number of photons reaching the detector) decreases as 1 / *q*.

The second protocol allows Bob to send an authenticated quantum state to Alice. At Alice’s side, anything that passes through the pinholes must originate from Bob. The state must be known to Bob, so that he can prepare the optical routing parameters.

QSA-d can be operated in a massively parallel way. The degree of parallelism depends on the ratio of a verifier’s time interval between challenge pulses and the prover’s time needed to switch optical paths. Parallel QSA-d has the potential to be much faster than cryptographic signing.

If Bob wants to authenticate a quantum state that is *unknown* to him, he has to adjust the optical routing using parameters $\alpha ,\beta$ that are ‘hidden’ inside a qubit. Such a feat requires a beam splitter controlled by the $(\alpha ,\beta )$ qubit, i.e., type of quantum gate or switch. This is a topic for future work.

It is interesting to compare QR-based authentication of quantum states to *cryptographic* authentication. Cryptographic authentication of a quantum message always requires encryption [1]. In our case the challenge state $| \psi \rangle$, which cannot be determined by the attacker, serves as a mask which hides $\alpha ,\beta$. In this respect the $\psi$ can be considered to be an ‘encryption key’ in our quantum authentication scheme.

In the field of optical encryption [20] phase masks are a popular physical encryption device that can be secure under specific conditions [21]. However, in contrast to PUFs, a phase mask can be easily replicated.

One important use case for QR-d is that it can serve as an additional authentication factor on top of the classical cryptographic authentication in Quantum Key Distribution. QKD then has two-factor authentication, where the two factors are entirely different. Furthermore, if the classical authentication key gets stolen (which typically is not noticeable), Alice and Bob still have the PUF-based authentication. Theft of the PUF does not go unnoticed.

It is interesting to note that physical location can also be used as an authentication factor [9]. Here the security is based on the assumption that the adversary has access only to a limited number of EPR pairs. Location as a credential can be used in combination with QR-d.

## Footnotes

1 For an introduction to post-quantum cryptography we refer to [2, 5].

2 For an overview on quantum cryptography and quantum communication in general see, e.g., [7, 14].

3 This is a security assumption referred to as *physical unclonability*. It cannot be mathematically proven. Whether the assumption holds or not depends on the technological state of the art.

4 The setup in [16] took much longer, but that was without any optimization for speed.
